# Beyond Pigmentation: Interplay of Metabolic Profile, Carotid Intima—Media Thickness, and Fatty Liver in Vitiligo: An Age‐ and Sex‐Matched Case‐Control Study

**DOI:** 10.1002/hsr2.73373

**Published:** 2026-10-22

**Authors:** Mehrdad Mozafar, Yasamin Bigdeli, Mehdi Azizmohammad Looha, Reza Samiee, Safoura Shakoei, Ifa Etesami, Zeinab Aryanian, Faezeh Khorasanizadeh, Parvaneh Hatami

**Affiliations:** ^1^ Autoimmune Bullous Disease Research Center, Razi Hospital Tehran University of Medical Sciences Tehran Iran; ^2^ Department of Surgery Division of Vascular and Endovascular Surgery, Shohada‐Tajrish Medical Center Shahid Beheshti University of Medical Sciences Tehran Iran; ^3^ School of Medicine Tehran University of Medical Sciences Tehran Iran; ^4^ Student Research Committee, Faculty of Medicine Qom University of Medical Sciences Qom Iran; ^5^ Basic and Molecular Epidemiology of Gastrointestinal Disorders Research Center, Research Institute for Gastroenterology and Liver Diseases Shahid Beheshti University of Medical Sciences Tehran Iran; ^6^ Department of Dermatology Imam Khomeini Complex Hospital Tehran University of Medical Sciences Tehran Iran; ^7^ Department of Dermatology Babol University of Medical Sciences Babol Iran; ^8^ Advanced Diagnostic and Interventional Radiology Research Center (ADIR) Tehran University of Medical Sciences Tehran Iran; ^9^ Department of Radiology Razi Hospital Tehran University of Medical Sciences Tehran Iran

**Keywords:** atherosclerosis, carotid intima–media thickness, metabolic syndrome, non‐alcoholic fatty liver disease, ultrasonography, vitiligo

## Abstract

**Background and Aims:**

Vitiligo shares inflammatory and metabolic pathways with cardiovascular and hepatic steatosis. Carotid intima–media thickness (IMT) is an established marker of early subclinical atherosclerosis, whereas non‐alcoholic fatty liver disease (NAFLD) reflects hepatic involvement in chronic inflammatory and metabolic dysregulation. This study aimed to evaluate subclinical vascular and hepatic changes in patients with vitiligo and to explore their associations with disease characteristics and metabolic parameters.

**Methods:**

In this age‐ and sex‐matched case‐control study, adults with mild vitiligo (Vitiligo Area Severity Index [VASI] < 5%) and no metabolic syndrome were compared with healthy controls. Participants underwent carotid duplex ultrasonography to assess common carotid IMT and atherosclerotic plaques, liver ultrasonography for steatosis grading, and laboratory testing, including lipid profile (total cholesterol, low‐density lipoprotein [LDL], high‐density lipoprotein [HDL], and triglycerides [TG]), fasting blood glucose (FBS), and body mass index (BMI). Disease duration and VASI score were also assessed.

**Results:**

A total of 46 participants (23 patients with vitiligo and 23 healthy controls) completed all assessments. Common carotid IMT measurements and liver ultrasound findings were comparable between groups. However, abnormal HDL levels were more frequent among patients with vitiligo (69.6% vs. 39.1%, *p* = 0.03). Greater disease severity, measured by VASI score, and higher BMI were independently associated with increased right carotid IMT (*p* = 0.01, 0.02, respectively). Exploratory interaction analyses demonstrated significant sex‐dependent associations of HDL, triglycerides, and fatty liver status with carotid IMT. Post hoc analyses identified higher carotid IMT among male patients with abnormal HDL.

**Conclusion:**

Selected subgroups of patients with vitiligo, particularly men with HDL metabolic abnormalities, may exhibit differences in subclinical carotid atherosclerosis. Disease severity and BMI may also contribute to the vascular risk. Although exploratory, these findings warrant further investigation of cardiovascular and hepatic risk profiles in selected patients with vitiligo.

## Key Messages

- Vitiligo may be associated with systemic inflammation, metabolic abnormalities, and increased cardiovascular risk.
- Patients with vitiligo had a higher prevalence of abnormal HDL levels than healthy controls.
- Exploratory analyses identified sex‐specific associations between metabolic abnormalities (abnormal HDL, elevated triglycerides, and fatty liver) and greater carotid intima–media thickness (IMT) in vitiligo patients, with the strongest evidence observed for abnormal HDL in male patients.
- Elevated vitiligo severity and higher BMI were also associated with increased carotid IMT.
- Early cardiovascular and hepatic risk assessment may help identify selected patients with vitiligo at increased cardiometabolic risk.

**Abbreviations**
ALT: alanine aminotransferase
ART: aligned rank transform
AST: aspartate aminotransferase
BMI: body mass index
CRP: C‐reactive protein
FBS: fasting blood sugar
FDR: false discovery rate
HDL: high‐density lipoprotein
IMT: intima‐media thickness
LDL: low‐density lipoprotein
MI: myocardial infarction
NAFLD: non‐alcoholic fatty liver disease
OR: odds ratio
TG: triglycerides
VASI: vitiligo area severity index

## 1 Introduction

Vitiligo is a chronic autoimmune skin disorder characterized by immune‐mediated destruction of melanocytes, resulting in sharply demarcated depigmented macules and patches that commonly involve the face, neck, and scalp [1]. It affects approximately 0.5%–2% of the global population, with a reported prevalence of 0.6% in Iran [2, 3]. Diagnosis is primarily clinical, with skin biopsy reserved for atypical or uncertain cases [4]. Disease extent and severity are commonly quantified using the vitiligo area severity index (VASI), a validated clinical scoring system widely used in both research and practice [5].

Vitiligo frequently co‐occurs with other autoimmune or chronic illnesses, including diabetes, thyroid disease, Addison's disease, and pernicious anemia [4]. It also shares inflammatory and metabolic pathways with metabolic syndrome [6], thereby predisposing affected individuals to cardiometabolic complications such as atherosclerosis [7]. Proinflammatory mediators such as IL‐1, IL‐6, and TNF‐α have been implicated in both conditions [7]. Several reports describe unfavorable metabolic profiles in vitiligo patients, including higher levels of triglycerides, cholesterol, Low‐Density Lipoprotein (LDL), fasting blood glucose (FBS), and high blood pressure compared with healthy controls [6, 8, 9, 10].

Carotid intima–media thickness (IMT), assessed by ultrasound, is a validated marker of subclinical atherosclerosis, with values > 0.8 mm indicating early vascular changes [11, 12]. Evidence from other chronic inflammatory dermatoses, such as psoriasis and lichen planus demonstrate increased carotid IMT, reinforcing the role of systemic inflammation in vascular damage [13, 14]. Non‐alcoholic fatty liver disease (NAFLD), likewise reflects systemic metabolic dysfunction and chronic inflammation and is independently associated with increased cardiovascular risk [15]. Given the shared inflammatory and metabolic pathways linking vitiligo, atherosclerosis, and NAFLD, evaluation of both carotid IMT and hepatic steatosis may provide complementary insights into early cardiometabolic involvement in patients with vitiligo.

Despite these overlaps, few studies have examined carotid IMT in vitiligo, and none have incorporated hepatic ultrasonography. This study therefore aimed to evaluate carotid IMT and liver steatosis in patients with vitiligo, and to explore their association with disease characteristics and metabolic profile. Establishing such links may help identify individuals at higher risk of cardiovascular events and NAFLD, supporting early preventive strategies.

## 2 Materials and Methods

### 2.1 Study Population

This exploratory observational case–control study included two groups of participants. The study commenced with sample collection from the phototherapy clinic and healthcare staff at Razi Hospital, Tehran, Iran. The patient group consisted of adults with non‐segmental vitiligo who attended the phototherapy clinic at Razi Hospital. The control group consisted of healthy volunteers recruited from nurses and other hospital staff at Razi Hospital during the same recruitment period. Controls were selected to be comparable to the patient group with respect to age and sex and were screened using the same inclusion and exclusion criteria, except for the absence of vitiligo. The sampling period lasted from November 2022 to August 2023. The study protocol was reviewed and approved by the Ethics Committee of Tehran University of Medical Sciences (Approval ID: IR.TUMS.MEDICINE.REC.1400.1028). All procedures involving human participants were conducted in accordance with the ethical standards of the institutional and/or national research committee and with the 1975 Helsinki Declaration, as revised in 1983. Written informed consent was obtained from all participants prior to enrollment. This study was reported in accordance with the Strengthening the Reporting of Observational Studies in Epidemiology (STROBE) Statement [16].

As this was an exploratory observational case–control study, no formal a priori sample size calculation was performed. Participant recruitment was based on the number of eligible individuals presenting to the phototherapy clinic during the predefined recruitment period (November 2022 to August 2023) who satisfied the eligibility criteria and completed all study assessments.

### 2.2 Inclusion and Exclusion Criteria

Eligible participants were adults (> 18 years) who provided informed consent to undergo carotid and hepatic ultrasonography as well as laboratory testing. Disease severity was evaluated using the VASI, with a VASI score < 5% classified as “mild,” consistent with established definitions of mild or localized vitiligo involving < 5%–10% of the body surface area [17]. Only patients with mild disease were included to minimize potential confounding from extensive inflammation or systemic oxidative stress, which could independently influence vascular or hepatic parameters [18]. This approach also reflects the clinical reality, as mild cases represent the majority of patients presenting to dermatology clinics and allow investigation of early, subclinical cardiometabolic alterations before advanced systemic involvement.

Participants with metabolic syndrome were excluded to specifically assess subclinical cardiometabolic changes potentially attributable to vitiligo‐related inflammatory mechanisms rather than those associated with established metabolic disease. This decision was based on previous evidence suggesting an increased prevalence of metabolic syndrome among patients with vitiligo [6], which may independently contribute to vascular and hepatic abnormalities. Inclusion of individuals with metabolic syndrome could therefore have introduced substantial confounding, as increased common carotid IMT and fatty liver changes are well‐documented in this population [19, 20]. Accordingly, the study aimed to evaluate the potential vasculometabolic effects of vitiligo in the absence of overt metabolic disease. Metabolic syndrome was defined as the presence of at least three of the following criteria [21]: triglycerides ≥ 150 mg/dL, HDL < 40 mg/dL (men) or < 50 mg/dL (women), fasting glucose ≥ 110 mg/dL, waist circumference ≥ 102 cm (men) or ≥ 88 cm (women), and blood pressure ≥ 130/85 mmHg. Because waist circumference measurement can be unreliable in some settings, a BMI ≥ 30 was used as a surrogate criterion [22]. Additional exclusion criteria were acute inflammatory conditions or infections, malignancy, heart failure, chronic liver or renal disease, thyroid dysfunction, hepatitis B or C, regular use of hepatotoxic medications (e.g., isoniazid, methotrexate, corticosteroids, amiodarone, tamoxifen), alcohol overuse (> 2 drinks/day for women, > 3 drinks/day for men), smoking, autoimmune or rheumatic conditions, immunodeficiency, immunosuppressant therapy, and a history of atherosclerotic disease, dyslipidemia, hypertension, diabetes, fatty liver disease, or pregnancy.

### 2.3 Data Collection Method

Eligible patients and controls were enrolled after confirmation of the inclusion and exclusion criteria and provision of written informed consent. Demographic information, including age, sex, education status, height, and weight (for BMI calculation) was collected from all participants. The duration of the disease was also recorded for the patient group, and the VASI score was calculated to determine disease severity. Participants were asked to undergo carotid and liver ultrasound and to undergo laboratory testing (FBS, Triglycerides (TG), total cholesterol, HDL, LDL, aspartate aminotransferase (AST), alanine aminotransferase (ALT), C‐reactive protein (CRP)) within a week of the ultrasound for more comprehensive analysis. Ultrasound examinations assessed the IMT of the common carotid arteries on both sides, the number of atherosclerotic plaques on each side, and the presence and grade of fatty liver in both groups. Laboratory and clinical findings were subsequently reviewed to confirm that participants did not meet the predefined diagnostic criteria for metabolic syndrome.

### 2.4 Carotid and Liver Ultrasound Evaluation

A high‐resolution B‐mode duplex ultrasonography system (Samsung Medison, Sonoace X8, Gyeonggi‐do, South Korea) with a 7.5–10 MHz linear array transducer and a minimum axial resolution of 0.3 mm was used to evaluate the IMT. An expert radiologist who was blinded to the subjects' grouping category conducted the measurements. All participants were examined while in a supine position, with their necks extended and their heads slightly tilted away from the side being examined. The IMT of the common carotid artery was defined as the distance between the lumen‐intima interface and the media‐adventitia interface of the vessel. Bilateral common carotid artery IMT measurements (in millimeters) were obtained within a 1 cm segment proximal to its bifurcation. Each measurement was performed three times, and the mean IMT was calculated. Atherosclerotic plaque was defined as a prominent protrusion into the vessel lumen with an IMT measure exceeding 1.2 mm. At the same session, liver ultrasound was performed to assess the presence of fatty liver and, when present, its grade. Liver ultrasound grading for NAFLD includes: 0) no evidence of fatty liver, (1) diffuse increase in liver echogenicity with normal periportal and diaphragm echogenicity, (2) diffuse increase in liver echogenicity obscuring periportal echogenicity but with normal diaphragm echogenicity, and (3) diffuse increase in liver echogenicity obscuring both periportal and diaphragm echogenicity [23].

### 2.5 Laboratory Data

Blood samples were collected from consenting participants at the laboratory department of Razi Hospital. The laboratory analyses included FBS, as well as TG, total cholesterol, HDL, and LDL, to evaluate metabolic risk factors. AST and ALT levels were measured to rule out underlying liver conditions that could potentially influence the ultrasonography findings. CRP levels were measured to assess systemic inflammatory status and to help exclude participants with evidence of acute inflammatory conditions or infections. For statistical analysis, lipid profiles were categorized as normal or abnormal based on standard laboratory cutoff values: TG > 135 mg/dL, total cholesterol > 200 mg/dL, HDL < 35 mg/dL, and LDL > 130 mg/dL were classified as abnormal, indicative of dyslipidemia. These thresholds were used to provide standardized definitions of dyslipidemia for statistical analyses.

### 2.6 Data Analysis

The Shapiro–Wilk test was used to assess the normality of continuous variables. Continuous variables are presented as mean ± standard deviation (SD) or median (interquartile range [IQR]), as appropriate according to their distribution. Categorical variables are presented as frequency (%). Group comparisons were performed using the independent‐samples t‐test or Mann–Whitney U test for continuous variables. Associations between categorical variables were evaluated using the Chi‐square test or Fisher's exact test, as appropriate. Separate multivariable linear regression models were fitted for each metabolic parameter, VASI, disease duration, and carotid IMT, with adjustment for age and sex. Regression coefficient (*β*) and adjusted coefficient of determination (adjusted *R* ^2^) were reported for each model. Subgroup analyses according to abnormal metabolic profiles and fatty liver status, as well as interaction analyses using aligned rank transform (ART) ANOVA [24], were conducted as exploratory analyses. The ART ANOVA was used to assess the main and interaction effects of categorical demographic variables, metabolic variables, and fatty liver status on carotid IMT among patients with vitiligo. Pairwise comparisons were performed using Dunn's post hoc test with false discovery rate (FDR) correction to account for multiple comparisons. All statistical analyses were performed using R software (version 4.4.2), and two‐sided *P* values < 0.05 were considered statistically significant.

## 3 Results

### 3.1 Study Population and Baseline Characteristics

A total of 78 individuals (42 patients with vitiligo and 36 healthy controls) met the eligibility criteria and underwent carotid and liver ultrasound evaluation. Nineteen patients with vitiligo and six healthy controls were subsequently excluded because they did not complete the laboratory assessment. None of the remaining participants met the diagnostic criteria for metabolic syndrome, and all satisfied the predefined inclusion and exclusion criteria. Following age‐ and sex‐matching, 23 vitiligo patients and 23 healthy controls were ultimately included in the study. Baseline demographic, laboratory, and ultrasonographic characteristics are summarized in Table 1. The mean age was 37.2 years for the patient group and 38.2 years for the healthy control group, with both groups including six male participants. The median duration of vitiligo in the patient group was 10 years, with a median disease severity of 1.75 based on the VASI core. Among laboratory parameters, HDL levels were significantly higher in the control group (median, 50 mg/dL [IQR, 45–56]) than in the vitiligo group (median, 43 mg/dL [IQR, 37–50]; *p* = 0.01).

**Table 1:** Demographic and clinical characteristics of the study.

Variable		Vitiligo patients (*N* = 23)	Control group (*N* = 23)	*p* value
Age (years)		37.22 ± 7.73	38.22 ± 8.24	0.67
Sex (Male)		26.1% (6)	26.1% (6)	> 0.99
Graduation	Undergraduate	13% (3)	0% (0)	0.20
	Diploma	60.9% (14)	47.8% (11)	
	Associate degree	8.7% (2)	13% (3)	
	Bachelor's degree	13% (3)	34.8% (8)	
	Master's degree	4.3% (1)	4.3% (1)	
Disease duration (years)		10 [5–17]	—	—
VASI (%)		1.75 [1–2.75]	—	—
BMI		25.27 ± 3.01	25.49 ± 3.79	0.95
Laboratory data	FBS (mg/dL)	92.04 ± 6.24	93 ± 7.21	0.63
	TG (mg/dL)	105.21 ± 38.61	105.34 ± 38.93	0.99
	Cholesterol (mg/dL)	159.52 ± 31.89	108.95 ± 35.03	0.41
	HDL (mg/dL)	43 [37–50]	50 [45‐56]	0.01*
	LDL (mg/dL)	92.32 ± 25.06	94.43 ± 29.76	0.76
	AST (U/L)	19.21 ± 5.52	19.78 ± 5.64	0.74
	ALT (U/L)	18.56 ± 10.4	19.34 ± 8.45	0.88
	CRP (mg/L)	3.2 [2.1–3.8]	2.7 [1.2–3.1]	0.06
Common carotid ultrasonography	Right Common Carotid IMT	0.5 [0.4–0.5]	0.5 [0.4–0.5]	0.22
	Left common carotid IMT	0.5 [0.4–0.5]	0.5 [0.4–0.5]	0.69
	Average bilateral common carotid IMT	0.45 [0.4–0.55]	0.45 [0.4–0.5]	0.93
	Presence of plaque in the right common carotid	0% (0)	0% (0)	—
	Presence of plaque in the left common carotid	0% (0)	8.7% (2)	0.48
Fatty liver ultrasonography	No fatty liver	73.9% (17)	73.9% (17)	> 0.99
	Grade 1 fatty liver	17.4% (4)	13% (3)	
	Grade 2 fatty liver	8.7% (2)	13% (3)	

*Note:* Normally distributed continuous variables are presented as mean ± standard deviation (SD), whereas non‐normally distributed variables are presented as median (interquartile range [IQR]). Categorical variables are presented as percentage (frequency). Group comparisons were performed using the independent‐samples *t* test or Mann–Whitney U test for continuous variables, and the Chi‐square test or Fisher's exact test for categorical variables, as appropriate.

Abbreviations: ALT; alanine aminotransferase, AST; aspartate aminotransferase, BMI; body mass index, CRP; C‐reactive protein, FBS; fasting blood sugar, HDL; high‐density lipoprotein, LDL; low‐density lipoprotein, VASI; vitiligo area scoring index, XTG; triglycerides.

Regarding ultrasound evaluations, the median right common carotid IMT was 0.5 mm (IQR, 0.4–0.5) in both the vitiligo and control groups (*p* = 0.22). Similarly, the median left common carotid IMT was 0.5 mm (IQR, 0.4–0.5) in both groups (*p* = 0.69), and the median average bilateral common carotid IMT was 0.45 mm (IQR, 0.4–0.55) in the vitiligo group and 0.45 mm (IQR, 0.4–0.5) in the control group (*p* = 0.93). No atherosclerotic plaques were detected in the right common carotid artery in either group. Left common carotid plaques were identified in two healthy controls (8.7%) and in no patients with vitiligo (*p* = 0.48). Most participants in both groups had no evidence of fatty liver on ultrasound (17/23 [73.9%] in each group). Grade 2 fatty liver was identified in 2/23 (8.7%) patients with vitiligo and 3/23 (13.0%) healthy controls.

To facilitate comparison of metabolic abnormalities between groups, laboratory variables were further categorized as normal or abnormal according to standard reference values (Figure 1). Overall, abnormal HDL levels were more common among patients with vitiligo than healthy controls (16/23 [69.6%] vs. 9/23 [39.1%], *p* = 0.03). In contrast, the control group had a higher prevalence of BMI > 30 (3/23 [13.0%] vs. 1/23 [4.3%], *p* = 0.61), abnormal total cholesterol (3/23 [13.0%] vs. 1/23 [4.3%], *p* = 0.61), and abnormal LDL levels (3/23 [13.0%] vs. 2/23 [8.7%], *P* = 1.00); however, none of these differences were statistically significant.

**Figure 1:**
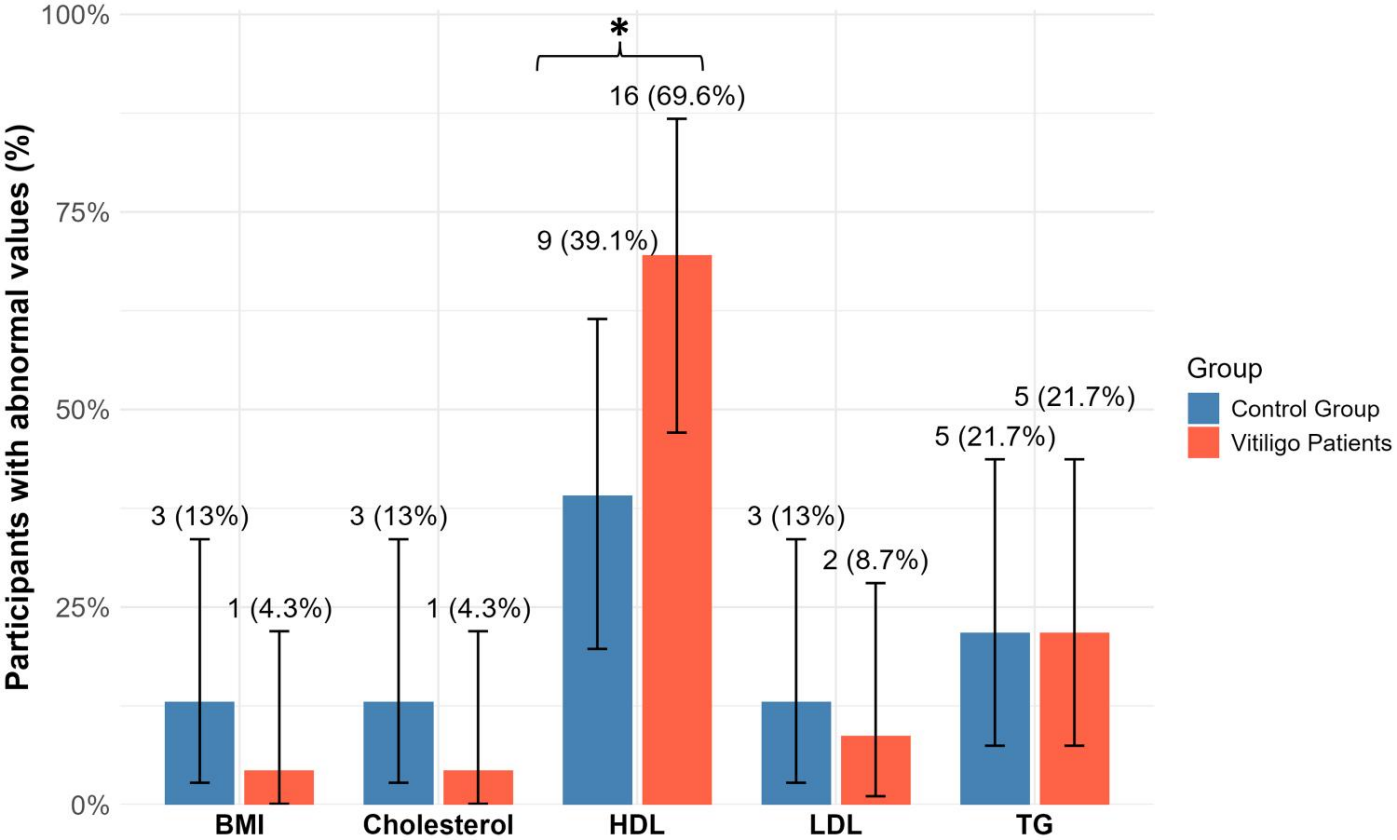
Frequency of abnormal metabolic profiles among patients with vitiligo and healthy controls. Bars represent the percentage of participants with abnormal metabolic parameters in each group. Error bars indicate the 95% confidence intervals for the estimated proportions. Numbers above the bars denote the number and percentage of participants with abnormal values. Abnormality thresholds were defined as: body mass index (BMI) ≥ 25 kg/m2, triglycerides (TG) > 135 mg/dL, high‐density lipoprotein (HDL) < 35 mg/dL, and low‐density lipoprotein (LDL) > 130 mg/dL. Abnormal fasting blood glucose (FBS > 110 mg/dL), aspartate aminotransferase (AST > 40 U/L), alanine aminotransferase (ALT > 40 U/L), and C‐reactive protein (CRP > 10 mg/L) were not included because no participant met these predefined abnormal thresholds. Group comparisons were performed using Fisher's exact test. ALT, alanine aminotransferase; AST, aspartate aminotransferase; BMI, body mass index; CRP, C‐reactive protein, FBS, fasting blood glucose; HDL, high‐density lipoprotein; LDL, low‐density lipoprotein; TG, triglycerides.

### 3.2 Clinical and Metabolic Determinants of Carotid IMT in Patients With Vitiligo

#### 3.2.1 Clinical Determinants of Carotid IMT

Disease duration and VASI score were examined in relation to common carotid IMT using multivariable linear regression models adjusted for age and sex (Figure 2). A significant positive association was observed between VASI score and right common carotid IMT (*β* = 2.2, adjusted *R* ^2^ = 0.24, *p* = 0.01), indicating that greater vitiligo severity was associated with increased right common carotid IMT. In contrast, VASI score was not significantly associated with left common carotid IMT (*β* = −0.13, adjusted *R* ^2^ = 0.06, *p* = 0.88) or average bilateral common carotid IMT (*β* = 1.03, adjusted *R* ^2^ = 0.14, *p* = 0.14). Likewise, disease duration was not significantly associated with right (*β* = −0.0001, adjusted *R* ^2^ = 0.24, *p* = 0.96), left (*β* = −0.002, adjusted *R* ^2^ = 0.06, *p* = 0.3), or average bilateral common carotid IMT (*β* = −0.001, adjusted *R* ^2^ = 0.14, *p* = 0.45).

**Figure 2:**
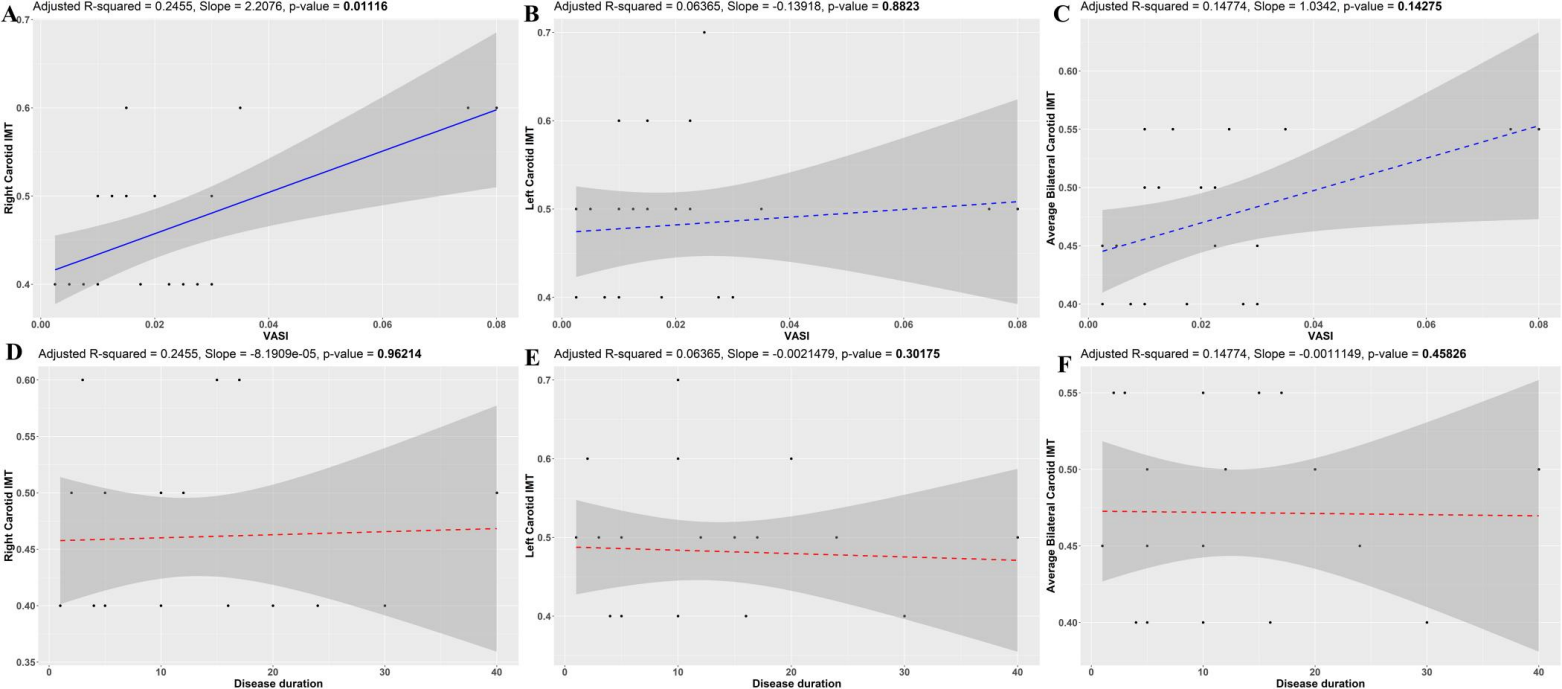
Associations between vitiligo area severity index (VASI) and disease duration with carotid intima‐media thickness (IMT) in patients with vitiligo. Panels show the relationship between VASI and (A) right carotid IMT, (B) left carotid IMT, and (C) average bilateral carotid IMT; and between disease duration and (D) right carotid IMT, (E) left carotid IMT, and (F) average bilateral carotid IMT. Linear regression lines are shown with shaded 95% confidence intervals. The top of each panel reports the adjusted *R* ^2^, slope (β), and P value from the corresponding multiple linear regression model adjusted for age and sex. Solid lines indicate significant associations, and dashed lines indicate non‐significant associations. Abbreviations: IMT, intima‐media thickness, VASI, vitiligo area severity index.

#### 3.2.2 Metabolic Determinants of Carotid IMT

The associations between continuous metabolic, and laboratory variables and common carotid IMT measurements are summarized in Table 2. Among patients with vitiligo, BMI was positively associated with right common carotid IMT (*β* = 0.46, *p* = 0.02). No statistically significant associations were observed between other laboratory variables, including FBS, triglycerides, total cholesterol, HDL, LDL, AST, ALT, or CRP, and right, left, or average bilateral common carotid IMT in the vitiligo group (all *p* > 0.05).

**Table 2:** Multiple linear regression analyses evaluating the associations between continuous metabolic parameters and carotid intima–media thickness (IMT).

Variables		Vitiligo group		Control group	
		Standardized regression coefficients (β)	*p* value	Standardized regression coefficients (β)	*p*value
BMI	Right carotid IMT	0.46	0.02*	−0.14	0.51
	Left carotid IMT	−0.10	0.46	−0.26	0.28
	Average bilateral IMT	0.18	0.38	−0.21	0.36
FBS	Right carotid IMT	0.27	0.20	−0.14	0.49
	Left carotid IMT	0.28	0.16	−0.01	0.98
	Average bilateral IMT	0.35	0.07	−0.07	0.74
TG	Right carotid IMT	0.03	0.86	−0.21	0.24
	Left carotid IMT	0.08	0.68	−0.18	0.38
	Average bilateral IMT	0.08	0.70	−0.20	0.30
Total cholesterol	Right carotid IMT	0.26	0.28	−0.16	0.40
	Left carotid IMT	−0.01	0.98	−0.15	0.50
	Average bilateral IMT	0.15	0.50	−0.16	0.44
HDL	Right carotid IMT	0.01	0.98	−0.19	0.33
	Left carotid IMT	−0.10	0.62	0.07	0.76
	Average bilateral IMT	−0.06	0.75	−0.06	0.77
LDL	Right carotid IMT	0.16	0.49	−0.10	0.58
	Left carotid IMT	0.01	0.95	−0.13	0.54
	Average bilateral IMT	0.10	0.63	−0.12	0.55
AST	Right carotid IMT	−0.30	0.16	−0.17	0.38
	Left carotid IMT	−0.09	0.71	−0.15	0.49
	Average bilateral IMT	−0.20	0.25	−0.17	0.42
ALT	Right carotid IMT	−0.20	0.37	−0.23	0.21
	Left carotid IMT	0.26	0.39	−0.16	0.46
	Average bilateral IMT	0.00	0.98	−0.20	0.31
CRP	Right carotid IMT	−0.07	0.72	−0.13	0.46
	Left carotid IMT	0.15	0.46	−0.14	0.51
	Average bilateral IMT	0.05	0.80	−0.14	0.47

*Note:* Values represent standardized regression coefficients (*β*) from multiple linear regression models adjusted for age and sex. An asterisk (*) denotes statistical significance (*p* < 0.05).

Abbreviations: ALT; Alanine aminotransferase, AST; Aspartate aminotransferase, BMI; Body mass index, CRP; C‐reactive protein, FBS; Fasting blood sugar, HDL; High‐density lipoprotein, IMT; Intima‐media thickness, LDL; Low‐density lipoprotein, TG; Triglyceride.

As an additional exploratory analysis, common carotid IMT measurements were compared between patients with vitiligo and healthy controls stratified according to abnormal TG, abnormal HDL, and fatty liver status (Supporting Information S1: Table 1). Analyses involving abnormal total cholesterol, LDL, BMI, and FBS were not performed because the number of participants with abnormal values was insufficient for reliable statistical comparisons. Median average bilateral common carotid IMT was similar between the vitiligo and control groups among participants with abnormal TG (0.40 [IQR, 0.42–0.50] vs. 0.50 [IQR, 0.40–0.52] mm; *p* = 0.74), abnormal HDL (0.47 [IQR, 0.40–0.55] vs. 0.45 [IQR, 0.40–0.50] mm; *p* = 0.63), and fatty liver (0.52 [IQR, 0.47–0.55] vs. 0.50 [IQR, 0.45–0.53] mm; *p* = 0.50). Likewise, no statistically significant differences were observed for right or left common carotid IMT in any comparison (all *p* > 0.05).

To further investigate factors associated with common carotid IMT among patients with vitiligo, ART ANOVA was performed using dichotomized demographic (sex) and metabolic variables with sufficient observations for statistical analysis, including TG, HDL, and fatty liver status (Table 3). A significant interaction between sex and HDL status was observed for right common carotid IMT (*F* = 10.21, *p* < 0.01), left common carotid IMT (*F* = 5.39, *p* = 0.03), and average bilateral common carotid IMT (*F* = 21.92, *p* < 0.001). Significant interactions were also identified between sex and TG status (*F* = 9.44, *p* < 0.01) and between sex and fatty liver status (*F* = 5.64, *p* = 0.02) for right common carotid IMT. In addition, a significant main effect of sex was observed for right common carotid IMT in the model including fatty liver status (*F* = 4.48, *p* = 0.04). No other main or interaction effects reached statistical significance (all *p* > 0.05). Post hoc analyses with FDR correction revealed that male patients with abnormal HDL had significantly greater average bilateral common carotid IMT than both female patients with abnormal HDL and male patients with normal HDL (*p* = 0.03). Given the limited subgroup sizes, these findings should be interpreted as exploratory and hypothesis‐generating.

**Table 3:** Main and interaction effects of demographic, fatty liver and metabolic variables on carotid intima–media thickness (IMT) among patients with vitiligo assessed using aligned rank transform (ART) ANOVA.

Dependent variable	Main effect 1	Main effect 1_F statistic	Main effect 1_*p value*	Main effect 2	Main effect 2_F statistic	Main effect 2_*p value*	Interaction	Interaction F statistics	Interaction_*p value*
Right carotid IMT	Sex	2.12	0.16	TG	0.08	0.77	Sex: TG	9.44	< 0.01*
	Sex	2.85	0.10	HDL	0.23	0.63	Sex: HDL	10.21	< 0.01*
	Sex	4.48	0.04*	Fatty liver	3.08	0.09	Sex: Fatty liver	5.64	0.02*
	TG	0.43	0.51	HDL	0.56	0.46	TG: HDL	0.20	0.65
	TG	2.23	0.15	Fatty liver	0.65	0.42	TG: Fatty liver	1.44	0.24
	HDL	1.98	0.17	Fatty liver	0.87	0.36	HDL: Fatty liver	1.80	0.19
Left carotid IMT	Sex	0.29	0.59	TG	0.12	0.72	Sex: TG	0.79	0.38
	Sex	0.06	0.80	HDL	0.15	0.69	Sex: HDL	5.39	0.03*
	Sex	0.19	0.66	Fatty liver	0.62	0.43	Sex: Fatty liver	0.04	0.84
	TG	0.00	0.97	HDL	0.00	0.97	TG: HDL	1.39	0.25
	TG	0.09	0.76	Fatty liver	1.65	0.21	TG: Fatty liver	4.25	0.05
	HDL	0.11	0.74	Fatty liver	0.63	0.43	HDL: Fatty liver	1.75	0.20
Average bilateral carotid IMT	Sex	1.14	0.29	TG	0.23	0.63	Sex: TG	0.31	0.58
	Sex	0.72	0.40	HDL	1.38	0.25	Sex: HDL	21.92	< 0.001*
	Sex	0.31	0.57	Fatty liver	1.06	0.31	Sex: Fatty liver	1.21	0.28
	TG	0.00	0.96	HDL	0.02	0.88	TG: HDL	1.25	0.27
	TG	0.02	0.88	Fatty liver	2.12	0.16	TG: Fatty liver	0.50	0.48
	HDL	0.48	0.49	Fatty liver	3.70	0.06	HDL: Fatty liver	4.14	0.05

*Note:* Aligned rank transform (ART) ANOVA was used to evaluate the main and interaction effects of categorical demographic and metabolic variables on carotid IMT. An asterisk (*) denotes statistical significance (*p* < 0.05). Analyses for abnormal cholesterol, BMI, fasting blood glucose, and LDL were not performed because of the low prevalence of abnormal values among patients.

Abbreviations: HDL; high‐density lipoprotein, IMT; intima‐media thickness, TG; triglycerides.

### 3.3 Clinical and Metabolic Characteristics According to Fatty Liver Status in Vitiligo Patients

Patients with vitiligo were stratified according to fatty liver status to evaluate associations with disease characteristics and metabolic profiles (Supporting Information S1: Table 2). Analyses of abnormal total cholesterol, LDL, BMI, and FBS were not performed because the number of patients with abnormal values was insufficient for reliable statistical evaluation. Disease duration, VASI score, and the prevalence of abnormal HDL and TG levels did not differ significantly between patients with and without fatty liver (all *p* > 0.05). Median disease duration was 13.5 years (IQR, 8.5–22.75) among patients with fatty liver and 10 years (IQR, 5–16.5) among those without fatty liver (*p* = 0.39). Likewise, median VASI scores were 2.25 (IQR, 1.56–7.62) and 1.5 (IQR, 0.62–2.5), respectively (*p* = 0.10). Abnormal HDL was present in 66.7% and 70.6% of patients (*p* = 0.85), while abnormal TG levels were observed in 33.3% and 17.6% (*p* = 0.57), respectively.

## 4 Discussion

In this study, no significant differences were observed between patients with vitiligo and healthy controls in common carotid IMT, carotid plaque prevalence, or fatty liver prevalence. However, exploratory analyses suggested that greater vitiligo severity, higher BMI, and selected metabolic factors may influence carotid IMT within specific patient subgroups. In particular, abnormal HDL levels were more frequent among patients with vitiligo, and post hoc analyses demonstrated that male patients with abnormal HDL had greater carotid IMT than female patients with abnormal HDL and male patients with normal HDL. Significant interaction effects were also observed between sex and triglyceride status, and between sex and fatty liver status, indicating that the relationship between these metabolic factors and carotid IMT may differ by sex. Given the modest sample size, limited subgroup sizes, and exploratory nature of these subgroup and interaction analyses, these findings should be interpreted cautiously as hypothesis‐generating observations rather than definitive associations.

Previous studies have consistently demonstrated increased carotid IMT in several chronic inflammatory dermatoses, supporting the concept that persistent systemic inflammation contributes to early vascular remodeling. For example, El‐Mongi et al. reported increased carotid IMT in patients with psoriasis, a finding subsequently confirmed by a systematic review and meta‐analys [25, 26]. Similarly, studies in hidradenitis suppurativa have demonstrated increased carotid IMT and a higher prevalence of subclinical atherosclerosis compared with healthy controls [27, 28]. Aryanian et al. also reported increased carotid IMT and a greater prevalence of atherosclerotic plaques in patients with lichen planus and, notably, identified a positive association between disease severity and carotid IMT [13].

Consistent with these findings, our exploratory analyses likewise suggested that greater disease severity in vitiligo was associated with increased carotid IMT. To our knowledge, only one previous study has evaluated carotid IMT in patients with vitiligo [11]. They reported a higher prevalence of subclinical atherosclerosis among patients with vitiligo but, similar to our findings, observed no significant difference in mean carotid IMT between patients and healthy controls [11]. Importantly, unlike the present study, its cohort was not restricted to patients with mild vitiligo. This distinction is particularly relevant because their multivariable analysis identified disease severity, measured by the VASI score, as an independent predictor of subclinical atherosclerosis. Likewise, although our primary between‐group analyses were negative, our exploratory analyses demonstrated that greater vitiligo severity was associated with increased right common carotid IMT. Taken together, these findings suggest that disease severity may represent an important determinant of vascular involvement in vitiligo and may partially explain differences between studies. Because our cohort consisted exclusively of patients with mild vitiligo (VASI < 5%) and excluded individuals with metabolic syndrome, whereas disease severity and metabolic status were not restricted in the study by Namazi et al. [11], our findings should be interpreted within this specific clinical context. The absence of significant between‐group differences in carotid IMT or carotid plaque prevalence may reflect the relatively low inflammatory and metabolic burden of our study population rather than the absence of vascular alterations in patients with more extensive disease or established metabolic abnormalities. Nevertheless, given the exploratory nature of our analyses and the limited sample size, this hypothesis requires confirmation in larger studies including patients across the full spectrum of disease severity.

The potential biological link between vitiligo and subclinical vascular dysfunction likely stems from a shared chronic inflammatory environment. Vitiligo is characterized by systemic oxidative stress and the elevation of proinflammatory cytokines, such as TNF‐α, IL‐1, and IL‐6, which are also key drivers of early atherosclerosis [7]. These inflammatory pathways have also been implicated in endothelial dysfunction and vascular remodeling [29]. Although inflammatory and oxidative‐stress pathways were not directly characterized in the present study, this biological context provides a plausible framework for the observed association between disease severity and carotid IMT.

A similar biological rationale may explain the positive association between BMI and carotid IMT observed in our study. Increased adiposity is associated with chronic low‐grade inflammation, endothelial dysfunction, oxidative stress, and adverse vascular remodeling, all of which are recognized contributors to early atherosclerosis [30]. Even in the absence of metabolic syndrome, excess body weight may amplify the vascular effects of chronic inflammation in patients with vitiligo [31]. Although this association should be interpreted cautiously given the exploratory nature of our analyses, it supports the potential contribution of body weight to early vascular changes in this population.

From a metabolic perspective, our study demonstrated a significantly higher prevalence of abnormal HDL levels among patients with vitiligo. Namazi et al. similarly reported altered HDL levels in this population, although they did not examine the prevalence of abnormal HDL according to established laboratory thresholds [11]. Reduced HDL is a well‐recognized marker of increased cardiovascular risk and has been linked to chronic systemic inflammation and oxidative stress, both of which are implicated in the pathogenesis of vitiligo [32]. Furthermore, in inflammatory states, HDL can lose its antioxidant and anti‐inflammatory properties, a phenomenon known as ‘dysfunctional HDL’, thereby impairing cholesterol efflux and promoting lipid peroxidation within the vessel wall [33]. Although our primary analyses did not demonstrate increased carotid IMT at the group level, the higher prevalence of abnormal HDL among vitiligo patients and the exploratory finding of increased carotid IMT in male patients with abnormal HDL suggest that vitiligo‐related systemic inflammation may accelerate vascular remodeling through both direct cytokine‐mediated damage and indirect metabolic dysregulation. These findings support further investigation in larger, adequately powered prospective studies to clarify whether lipid abnormalities contribute to vascular alterations in selected patients with vitiligo and whether such associations have any implications for cardiovascular risk stratification.

The prevalence of fatty liver did not differ significantly between patients with vitiligo and healthy controls in the present study. To our knowledge, this is the first study to evaluate hepatic steatosis using ultrasonography in patients with vitiligo. In contrast, an increased prevalence of fatty liver has been reported in other chronic inflammatory dermatoses, including hidradenitis suppurativa and psoriasis [34, 35, 36]. The absence of a significant difference in our cohort may reflect the exclusion of individuals with metabolic syndrome and the inclusion of only patients with mild vitiligo, thereby minimizing the influence of established metabolic risk factors. Alternatively, hepatic involvement in vitiligo may be less pronounced than in other chronic inflammatory dermatoses or may become evident only in patients with more extensive disease or greater metabolic burden. Larger prospective studies including patients across the full spectrum of disease severity are needed to clarify the relationship between vitiligo and fatty liver disease.

From a clinical perspective, our findings do not support routine carotid ultrasonography or hepatic imaging for all patients with vitiligo. Instead, these results raise the possibility that selected patients with greater disease severity, obesity, or metabolic abnormalities such as reduced HDL levels may represent subgroups requiring more careful assessment of cardiovascular risk factors. At present, the cost‐effectiveness and clinical utility of targeted vascular or hepatic imaging in vitiligo remain uncertain. Future prospective studies should determine whether specific clinical characteristics can identify patients who may benefit from additional screening beyond standard cardiovascular risk assessment.

### 4.1 Strengths and Limitations

By excluding individuals with metabolic syndrome and focusing on patients with mild vitiligo, this study specifically examined the vasculometabolic aspects of vitiligo while minimizing important metabolic confounding. Mild vitiligo represents a common phenotype encountered in clinical practice and may provide an opportunity to investigate early subclinical cardiometabolic alterations. However, these selection criteria also limit the external validity of our findings. Therefore, the findings should not be extrapolated to individuals with moderate‐to‐severe disease, extensive inflammatory burden, or established metabolic abnormalities. Although this study design and the strict control of important confounding factors strengthen the internal validity of the findings, they also contributed to the modest sample size. In addition, participant recruitment was limited to eligible individuals presenting during the predefined study period, and participant withdrawal during follow‐up further reduced the final sample size. Consequently, the study may have been underpowered to detect small or moderate between‐group differences, particularly in subgroup and interaction analyses. These findings should therefore be regarded as exploratory and interpreted with caution until replicated in larger cohorts. In addition, the cross‐sectional design limits the ability to establish temporal relationships between vitiligo severity, metabolic abnormalities, and vascular changes. Longitudinal studies with larger cohorts and extended follow‐up are required to determine whether carotid IMT progression or other subclinical vascular markers predict future cardiovascular events in patients with vitiligo.

Despite these limitations, the study has several important strengths, including its rigorous methodology, strict control of major metabolic confounders, comprehensive ultrasonographic evaluation of both the carotid arteries and liver, and the simultaneous assessment of vascular, hepatic, and metabolic parameters within a well‐characterized cohort of patients with vitiligo.

## 5 Conclusions

In conclusion, no significant differences were observed between patients with vitiligo and healthy controls in carotid IMT, carotid plaque prevalence, or fatty liver prevalence. However, exploratory analyses suggested that greater disease severity, higher BMI, and selected metabolic abnormalities may be associated with increased carotid IMT. In particular, male patients with abnormal HDL demonstrated greater carotid IMT, while interaction analyses suggested that sex may modify the associations of triglyceride levels and fatty liver status with carotid IMT. These findings should be interpreted cautiously because of the modest sample size and exploratory subgroup analyses. They do not support routine vascular or hepatic imaging in patients with mild vitiligo but highlight the need for larger prospective studies to determine whether selected patients with greater disease severity or metabolic risk factors may benefit from targeted cardiovascular risk assessment.

## Author Contributions

**Mehrdad Mozafar:** conceptualization; formal analysis; writing – original draft; writing – review and editing; project administration; supervision; investigation; methodology; validation. **Yasamin Bigdeli:** conceptualization; writing – review and editing; writing – original draft. **Mehdi Azizmohammad Looha:** visualization; writing – review and editing; validation. **Reza Samiee:** writing – original draft; writing – review and editing; data curation. **Safoura Shakoei:** conceptualization; visualization; supervision; writing – review and editing; validation. **Ifa Etesami:** conceptualization; writing – review and editing; project administration; supervision; validation. **Zeinab Aryanian:** conceptualization; formal analysis; funding acquisition; validation; project administration; writing – review and editing. **Faezeh Khorasanizadeh:** conceptualization; project administration; writing – review and editing; funding acquisition; validation. **Parvaneh Hatami:** conceptualization; methodology; project administration; validation; writing – review and editing.

## Disclosure

Zeinab Aryanian and Faezeh Khorasanizadeh affirm that this manuscript is an honest, accurate, and transparent account of the study being reported; that no important aspects of the study have been omitted; and that any discrepancies from the study as planned (and, if relevant, registered) have been explained. All authors have read and approved the final version of the manuscript. Zeinab Aryanian and Faezeh Khorasanizadeh, the corresponding authors and manuscript guarantors, had full access to all of the data in this study and takes complete responsibility for the integrity of the data and the accuracy of the data analysis.

## Ethics Statement

This study was conducted in accordance with the ethical standards of the institutional and national research committees and with the 1964 Declaration of Helsinki and its later amendments. Ethical approval was obtained from the Ethics Committee of Tehran University of Medical Sciences (approval number: IR.TUMS.MEDICINE.REC.1402.123). All study procedures, including participant recruitment, clinical assessments, and laboratory and ultrasonographic evaluations, were reviewed and approved by the committee prior to initiation.

## Consent

Written informed consent was obtained from all participants before enrollment. Participants were informed of the study objectives, procedures, and their right to withdraw at any stage without any impact on their medical care.

## Conflicts of Interest

The authors declare no conflicts of interest.

## Supporting information


Supporting File 1

Supporting File 2

## Data Availability

The research data supporting this study are available from the corresponding author upon reasonable request.
