# Supplementary material for: Beyond Pigmentation: Interplay of Metabolic Profile, Carotid Intima—Media Thickness, and Fatty Liver in Vitiligo: An Age‐ and Sex‐Matched Case‐Control Study
Source: Health Sci Rep. 2026 Oct 22;9(11):e73373. doi: 10.1002/hsr2.73373 (PMC13691529; doi:10.1002/hsr2.73373)
Supplement: Supplementary file 1 [file HSR2-9-e73373-s001.docx]

**Supplementary Table 2.** Comparison of disease characteristics and metabolic profiles according to fatty liver status among patients with vitiligo.

| Liver ultrasound assessment | Disease duration | | VASI | | Abnormal HDL | | Abnormal TG | |
| --- | --- | --- | --- | --- | --- | --- | --- | --- |
|  | **Median (IQR)** | **P value** | **Median (IQR)** | **P value** | **Frequency (%)** | **P value** | **Frequency (%)** | **P value** |
| Presence of Fatty Liver (N=6) | 13.5 (8.5-22.75) | 0.39 | 2.25 (1.56-7.62) | 0.10 | 4 (66.6%) | 0.85 | 2 (33.3%) | 0.57 |
| Absence of Fatty Liver (N=17) | 10 (5-16.5) |  | 1.5 (0.62-2.5) |  | 12 (70.58%) |  | 3 (17.64%) |  |

Continuous variables are presented as median (interquartile range [IQR]), and categorical variables as frequency (%). Disease duration and VASI scores were compared using the Mann–Whitney U test. Categorical variables (abnormal HDL and abnormal TG) were compared using Fisher's exact test. Analyses for abnormal cholesterol, BMI, fasting blood glucose (FBS), and LDL were not performed because of the low prevalence of abnormal values among patients. **Abbreviations:** IQR; Interquartile range, VASI; Vitiligo Area Scoring Index, BMI; Body Mass Index, FBS; Fasting Blood Sugar, TG; Triglycerides, HDL; High-Density Lipoprotein, LDL; Low-Density Lipoprotein.
