# Supplementary material for: Beyond Pigmentation: Interplay of Metabolic Profile, Carotid Intima—Media Thickness, and Fatty Liver in Vitiligo: An Age‐ and Sex‐Matched Case‐Control Study
Source: Health Sci Rep. 2026 Oct 22;9(11):e73373. doi: 10.1002/hsr2.73373 (PMC13691529; doi:10.1002/hsr2.73373)
Supplement: Supplementary file 2 [file HSR2-9-e73373-s002.docx]

**Supplementary Table 1.** Comparison of carotid intima–media thickness (IMT) between patients with vitiligo and healthy controls stratified by abnormal metabolic profiles and fatty liver status.

| Variables | Study group | Right Carotid IMT | | Left Carotid IMT | | Average Bilateral Carotid IMT | |
| --- | --- | --- | --- | --- | --- | --- | --- |
|  |  | **Median (IQR)** | **P value** | **Median (IQR)** | **P value** | **Median (IQR)** | **P value** |
| Abnormal TG | **Vitiligo (n=5)** | 0.4 (0.4-0.5) | 0.54 | 0.5 (0.4-0.5) | 0.72 | 0.4 (0.42-0.5) | 0.74 |
|  | **HC (n=5)** | 0.5 (0.4-0.5) |  | 0.5 (0.4-0.6) |  | 0.5 (0.4-0.52) |  |
| Abnormal HDL | **Vitiligo (n=16)** | 0.5 (0.4-0.5) | 0.54 | 0.4 (0.4-0.5) | 0.49 | 0.47 (0.4-0.55) | 0.63 |
|  | **HC (n=9)** | 0.4 (0.4-0.57) |  | 0.5 (0.4-0.5) |  | 0.45 (0.4-0.5) |  |
| Presence of Fatty liver | **Vitiligo (n=6)** | 0.5 (0.4-0.6) | 0.71 | 0.5 (0.47-0.55) | 0.65 | 0.52 (0.47-0.55) | 0.50 |
|  | **HC (n=6)** | 0.5 (0.5-0.52) |  | 0.5 (0.4-0.55) |  | 0.5 (0.45-0.53) |  |

Variables are presented as median (interquartile range [IQR]). Group comparisons were performed using the Mann–Whitney U test. Analyses for abnormal cholesterol, BMI, fasting blood glucose, and LDL were not performed because of the low prevalence of abnormal values in the study groups. **Abbreviations:** HC; Healthy Controls, IQR; Interquartile range, BMI; Body Mass Index, FBS; Fasting Blood Sugar, TG; Triglycerides, HDL; High-Density Lipoprotein, LDL; Low-Density Lipoprotein.
